# Opioid Use Disorder: Treatments and Barriers

**DOI:** 10.7759/cureus.13173

**Published:** 2021-02-06

**Authors:** Karan Patel, Sean Bunachita, Ank A Agarwal, Aaron Lyon, Urvish K Patel

**Affiliations:** 1 Medicine, Cooper Medical School, Camden, USA; 2 Medicine, Johns Hopkins University, Baltimore, USA; 3 Medicine, Brigham Young University-Idaho, Rexburg, USA; 4 Public Health and Neurology, Icahn School of Medicine at Mount Sinai, New York, USA

**Keywords:** opioids use disorder, pharmacological treatment, non-pharmacological treatment

## Abstract

Over the last decade, opioid use around the world has risen considerably and is projected to continue to rise at an alarming rate. As opioid use rises, so too does the number of people who suffer from opioid use disorder (OUD) and opioid overdose-related deaths. As science and medicine progresses, new medications and therapies have arisen in order to help treat patients suffering from addiction. Treatment can be split into two main domains: pharmacological and non-pharmacological. Buprenorphine and methadone, currently the most prescribed medications for patients suffering from OUD, have been shown to be extremely effective in clinical trials but have significant real-world limitations. Geographical disparities between various locations, physician stigma with prescribing these medications, and training required to prescribe medication can make access to these treatments difficult for patients. Non-pharmacological interventions have also been shown to help with limited efficacy when combined with pharmacological interventions. However, the time and resources required to implement these strategies may be a difficult barrier to overcome. In this review, we assess pharmacological interventions, non-pharmacological treatments, examine barriers to treatment for patients, and propose solutions to bypass these barriers.

## Introduction and background

Between 1999 and 2019, close to 450,000 people died from an opioid overdose [1]. Compared to 20 years ago, the death rate due to opioid overdoses is four times higher today. The increases in the number of overdoses have followed three major waves: a rise in prescription opioids, rises in the use of heroin, and the advent of other synthetic opioids (e.g. fentanyl) [2-4]. While the data from 2017 to 2018 actually showed a 4.1% decrease in overdoses, this was an anomaly in terms of the historical trends [5]. Between 2018 and 2019, there was a 4.6% increase in the number of total drug overdoses, with opioid overdoses accounting for 75% of these deaths [6]. These numbers are projected to spike in 2020-2021 due to the coronavirus pandemic creating extra barriers to receiving treatment. In the first three months of 2020, there were 3000 more overdose-related deaths compared to 2019 [7]. While this year may again be an extreme, there have been multiple models that have shown that the number of opioid-related deaths is expected to rise and is projected to reach 700,000 (82,500 a year) between the years 2016 and 2025; a substantial increase in numbers over the last two decades [8].

Geographical locations have also had an impact on the number of overdoses. For example, urban regions, such as medium-sized metropolitan areas (defined as a population between 250,000 to 999,999 in the United States), had the highest rates of opioid overdose deaths (5.9 per 100,000) followed by micropolitan areas (5.6 per 100,000) and non-metropolitan areas (5.3 per 100,000) [9]. It comes as no surprise that among those with the lowest number of opioid overdoses come in large and fringe metropolitan areas [9]. After all, people in these areas tend to live closer to hospitals and tend to have more physicians per square mile [7].

One of the most significant challenges comes in preventing opioid use/overdoses in rural areas and underdeveloped regions where there is reduced access to medical resources and addiction treatment centers [7]. While in this article, we focus primarily on opioid use in the United States, it is an interesting point to note that some underdeveloped countries with fewer treatment centers and access to medical care actually have fewer patients addicted to and overdosing on opioids. Although this could partially be attributed to differences in social-cultural perspectives on drug use, it may also simply be due to lack of access - for example, the United States accounted for 56% of global morphine consumption in 2009 [10].

## Review

Pharmacological treatments

There are four primary pharmacological treatments for opioid use/withdrawal. These include naloxone, naltrexone, buprenorphine, and methadone. While all of them are therapeutically effective and play a role in helping curb the epidemic, in this article, we focus on the latter two. Buprenorphine and methadone are currently the most widely prescribed pharmacological opioid treatments and have been shown to have the highest efficacy in clinical trials [11].

Buprenorphine

Buprenorphine, a synthetic derivative of thebaine, has served as the gold standard of treatment for OUD since its development in 1960. Outside of treating OUD, it has also been approved for the treatment of acute and chronic pain. However, we will limit our discussion to its use in treating opioid withdrawal. The drug is a partial agonist that has a high affinity but low dissociation to the mu-opioid receptor [12]. Because it works in a similar manner to opioids, the effects of buprenorphine and opioids overlap in that they include euphoria (based on the dosage) and miosis, but buprenorphine does not affect blood pressure, respiratory rate, or heart rate [13]. Out of the major categories of pharmacological cost treatments, buprenorphine is the most cost-effective, costing about 112 dollars per week or 6,000 dollars per year [14].

There are two main forms of buprenorphine, a sublingual and a subcutaneous form. The sublingual form is generally administered daily and is combined with naloxone in order to prevent patient abuse. Naloxone prevents patients from creating a liquid from the sublingual capsules that can then be used as an intravenous (IV) injection. Generally, in the first months of treatment, buprenorphine is only administered by pharmacists, and as a patient gradually gains the health care provider’s trust, they can receive doses to take home [15]. Overall, however, the abuse potential for buprenorphine is relatively low: the drug is classified by the Food and Drug Administration (FDA) as causing only mild physical dependence. The subcutaneous form has the same mechanism as the sublingual form, however, its main advantage is that it requires administration far less often (up to only once every six months) [16].

Clinical trials comparing buprenorphine vs. placebo has shown that the drug is extremely effective: at mild doses (2-6 mg/day), it has shown to help treatment retention in one-quarter of patients, at moderate doses (6-16 mg/day), that number rises to one-third of patients, and finally, at high doses (>16mg/day), that number further increases to one-half of patients [17]. As a comparison, statins, which are one of the most highly prescribed drugs, have a number needed to treat (NNT) of 227 for non-fatal strokes [18]. While some may argue that this is not a fair comparison in that one is talking about treatment retention and the other is talking about the direct number of lives saved, improvements in treatment retention allow for improved patient survival, decreased opioid use, improved birth outcomes in pregnant women, and higher job retention rates.

Methadone

Methadone, a long-acting full agonist of the mu-opioid receptor, has very similar effects to both morphine and heroin, including possessing analgesic properties and causing respiratory depression. However, it has a longer half-life, causes less intense withdrawal symptoms, and can block the euphoric effects of other opioids. In addition to its ability to activate the mu-opioid receptors, methadone has also been shown to be a potent antagonist of the N-methyl-D-aspartic acid (NMDA) receptor, which has been implicated in both neuropathic pain and opioid tolerance [19]. Contraindications of methadone include patients who have hypersensitivity reactions to methadone hydrochloride, as well as patients who have respiratory depression or are suspected to have a paralytic ileus [20]. However, it is safe for use in the vast majority of the population. The drug has to initially be taken under medical supervision until the patient gains the trust of the healthcare professional, after which they may prescribe doses to take at home.

Similar to buprenorphine, methadone too has been shown to be extremely effective from a clinical standpoint. A review article showed that data from clinical trials suggest that taking high doses of methadone (80-120mg) in patients who were once addicted to heroin, had up to an 80% success rate (endpoints based on social reintegration/productively and job reemployment) [21]. Furthermore, the cost of treatment, including biopsychosocial social services, is not too steep. It ranges around 6,500 dollars per year [14].

However, some of the primary issues associated with the drug are access and frequency of use. When starting treatment, methadone has to be taken every eight to 12 hours and should follow the course of at least 12 months, with many patients requiring longer treatment periods [22]. In addition, the drug can only be dispensed by Substance Abuse and Mental Health Services Administration (SAMHSA)-certified treatment programs [22]. This inherently creates a disparity in access since these programs are not evenly distributed throughout the country. As a result, a patient's geographical location will play a role in whether or not they are able to access treatment. Another issue with methadone comes not from the drug itself but from those who are prescribing the medication. Because addiction does not have the same presentations as any other disease many in the medical community still hesitate to treat it in the same manner they would any other disease - through medical intervention. Methadone becomes especially problematic in this regard since it is a full agonist and so it closely mimics drugs such as morphine or heroin. Many primary care physicians believe that in prescribing methadone all they are doing is “replacing one addiction with another” [23]. While some patients do become addicted to methadone, it may be a necessary step in order to resolve a patient's opioid addiction. After all, quitting methadone is far easier than quitting opioids since it causes less severe withdrawal symptoms [19].

Non-pharmacological treatments

Cognitive-Behavioral Therapy 

Cognitive-behavioral therapy (CBT) is a non-pharmacological intervention that has been shown to be highly effective in treating a number of psychological disorders, including depression, anxiety disorders, and substance use disorders [24]. As the name implies, CBT is centered on both cognitive and behavioral etiologies and approaches. On the cognitive end is the idea of motivational enhancement - focusing on increasing a patient’s motivation to modify their behaviors and adhere to treatments [25]. Such models target restructuring maladaptive thoughts and cognitive processes through methods ranging from motivational interviewing to acceptance and commitment therapy [26]. The behavioral portion of CBT is rooted in learning theory and is concentrated on identifying functional cues for opioid use and offering alternative, less destructive, responses [25,27]. One example is the relapse prevention (RP) approach, which includes determining factors that increase the risk of opioid usage (e.g. attending parties or having friends who also use opioids) and working to either prevent exposure to such situations or offering strategies to mitigate the potential of abuse [25]. Some techniques of RP are to educate the patient such that they can make more informed decisions in high-risk situations and to analyze the consequences and perceived benefits of opioid usage [25]. With respect to OUD, CBT, in combination with methadone maintenance treatment (MMT), has been shown to increase the length of abstinence from opiates and reduce psychological stress [28-29]. Further, while individual CBT in combination with buprenorphine showed no additive benefits, buprenorphine with group CBT demonstrated a statistically significant reduction in opioid use [26]. The greater effectiveness of group CBT may be due to its ability to foster a productive exchange of methods and experiences pertaining to OUD recovery and an environment that fosters discussion whereupon participants respectfully challenge peers in ways that may not be deemed appropriate or sincere between patient and clinician [26].

Contingency Management 

Contingency management (CM) is one of the most effective treatments for patients addicted to drugs, yet it is rarely used by healthcare providers [30]. It relies on using positive reinforcement models to reward patients who continuously produce negative drug tests. While there are a variety of CM models that have been developed, the most commonly used ones are voucher-based reinforcement and prize-based incentives. Voucher-based reinforcement is a strategy where each time a previously addicted patient produces a negative drug test, they are rewarded with a voucher that can then be redeemed for a tangible product such as food, goods, or services. Prize-based incentives also follow a similar model, but instead of the voucher, they give out monetary compensation. After a negative drug test, the patient then receives a bowl and picks out a slip from a pile labeled with various cash values. Depending on the slip they select, they are rewarded with an amount of money, which generally ranges from 1 to 100 dollars. As the patient continues to produce negative drug test results, they are allowed to have an increased number of draws [31]. One of the issues with contingency management is the lack of centers that provide this form of treatment. However, with the ever-expanding technological realm, contingency management models have now been developed for online use and, as a result, may be able to reach a greater population [32]. While accessing certain populations may no longer be a challenge for CM, having adequate resources still serves as the largest barrier. Because CM relies on having “rewards,” the question of who exactly will fund these rewards arises. Models in France and the UK have shown that community donations may be an effective solution as a primary source of funds. Research has shown that although CM may have a higher up-front initial cost, it may actually be more cost-effective in regard to patient outcomes over the long term [33].

Barriers to treatment/strategies to address barriers

Stigma

Despite the life-saving characteristics associated with the treatments proposed, a number of barriers can impede their adherence or accessibility to patients. One such obstacle is the stigma of healthcare professionals against OUD patients [34]. This type of stigma is particularly common: a systematic review found that health and social care workers generally held strongly negative attitudes toward patients with illicit substance use disorders [35]. These negative schemas have been discovered to partially arise from the perception that substance abuse patients are aggressive, manipulative, and unmotivated [36]. Moreover, substance use disorders are generally viewed as having high controllability; i.e., the use of illicit drugs is a conscious decision and thus the patient is perceived to have greater responsibility for their condition as opposed to disorders such as psychosis or AIDS [35]. The result of such stigma has the potential to lead to worse health outcomes for patients as a consequence of the suboptimal quality of care: for instance, nurses have been shown to make shorter visits and have a more impersonal, task-oriented approach when treating substance abuse patients [37]. Strategies that have been proposed to counteract stigma primarily focus on educating healthcare professionals about substance use disorders, as this has been found to promote more positive attitudes regarding addiction and greater optimism for treatment outcomes [38]. In addition, organizational and role support structures for health professionals working with substance use disorder patients have also been determined to be key factors that positively influence attitudes surrounding the disease [39].

Legal Restrictions for Pharmacological Treatments

Another obstruction of patient access to care are laws and regulations that limit or further burden healthcare providers wishing to prescribe pharmacological treatments. These restrictions can be federally or state-regulated: some examples of federal regulation are the 275-patient cap of buprenorphine imposed by the Drug Addiction and Treatment Act of 2000 and the inability of physicians outside of Opioid Treatment Program (OTP) clinics to prescribe methadone under the Narcotic Addict Treatment Act of 1974 [40-41]. At the state level are stipulations that require a prescription of buprenorphine to be paired with opioid dependence counseling and prevent primary care physicians from billing for substance use disorder treatment in non-specialty settings [34,42]. In order to address these limitations, it is important for public health leaders and mental health advocates to lobby for legislators to repeal or modify existing laws that serve to delay or prohibit accessibility. For instance, one study found that counseling added no benefit to the effectiveness of buprenorphine; treatment using the medication alone provided the same outcomes [42]. Further, expanding the ability of healthcare professionals to prescribe methadone outside of OTPs could bolster rates of treatment in rural areas, as 97% of counties in the United States with populations lower than 20,000 reported shortages in OTP availability [41].

Inadequate Training

Yet another barrier to treatment is insufficient training of healthcare professionals. Only a reported 6% of physicians in the United States have received the SAMHSA and Drug Enforcement Agency (DEA) authorization waiver required to prescribe buprenorphine, which has led to almost 50% of all US counties not having even one physician with buprenorphine authorization [43]. While the primary requirement to acquire the waiver is a course (8 hours long for physicians, 24 hours for nurse practitioners) that is free and relatively easy to access, it does create another hurdle to treatment. Additionally, a majority of physicians specializing in primary care and addiction cited inadequate knowledge, education, and experience in prescribing buprenorphine, with both medical school training and the waiver process seen as deficient in providing a suitable education of OUD and its associated treatments [44]. An approach that can be taken to address insufficient clinician training would be to provide more comprehensive opioid education and treatment experience in graduate-level studies. One study discovered that psychiatrists who had any form of buprenorphine training in residency were more likely to prescribe buprenorphine in later practice [45]. An added benefit to earlier, mandatory buprenorphine training is that it could eliminate the necessity of taking an additional course to obtain the authorization waiver, potentially increasing the number of physicians qualified to prescribe the medication.

## Conclusions

OUD continues to be a major concern in the United States, especially for those living in rural counties. While various pharmacological and non-pharmacological treatments are well-studied and can complement one another to form an effective treatment plan for patients, a number of barriers exist that can impact accessibility to care. These include stigma among healthcare professionals against OUD patients, numerous legal restrictions for pharmacological treatment, and inadequate training of clinicians. It is imperative that these impediments are addressed in a manner that will promote more widespread OUD treatment in order to curb the prevalence of OUD across the country. 
